# GLP-1 AGONISTS: EFFECT IN MANAGEMENT OF DIABETES AND WEIGHT LOSS IN OLDER ADULTS WITH OVERWEIGHT AND OBESITY

**DOI:** 10.1093/geroni/igad104.2256

**Published:** 2023-12-21

**Authors:** Anna Pendrey

**Affiliations:** Indiana University, Indianapolis, Indiana, United States

## Abstract

Prevalence of Diabetes Mellitus Type 2 in older adults in the US is 33%, incidence of newly diagnosed diabetes is highest among those aged 65-79 years. Obesity in older adults impacts not only the morbidity and mortality, but importantly impacts quality of life and risk of institutionalization. Treatment for diabetes with glucagon like peptide 1 (GLP-1) agonists have demonstrated to reduce weight, control glucose, and reduce major adverse cardiovascular events in older adults. This study aims to describe outcomes in the management of older adults with diabetes and obesity with GLP-1 agonists. A retrospective study of 30 older adults with uncontrolled diabetes, overweight and obesity at a local practice that were started on treatment with GLP-1 agonists, since 2022, with ages 65-84 with diabetes mellitus type 2 with initial HbA1C 9.6%-12.6%, and BMI 27-48.2 that were started on GLP-1 agonists with improved results during treatment with current reduction of HbA1C 5.8%-7.7% and BMI reduction 23-39.8. In conclusion GLP-1 agonists have demonstrated significantly improved management of uncontrolled Diabetes Mellitus in older adults as well as a significant reduction in weight improving outcomes.

